# Investigating Different Levels of Bimanual Interaction With a Novel Motor Learning Task: A Behavioural and Transcranial Alternating Current Stimulation Study

**DOI:** 10.3389/fnhum.2021.755748

**Published:** 2021-11-16

**Authors:** Marleen J. Schoenfeld, Ioana-Florentina Grigoras, Charlotte J. Stagg, Catharina Zich

**Affiliations:** ^1^Oxford Centre for Human Brain Activity, Wellcome Centre for Integrative Neuroimaging, Department of Psychiatry, University of Oxford, Oxford, United Kingdom; ^2^Wellcome Centre for Integrative Neuroimaging, FMRIB, Nuffield Department of Clinical Neurosciences, University of Oxford, Oxford, United Kingdom; ^3^Medical Research Council Brain Network Dynamics Unit, Nuffield Department of Clinical Neurosciences, University of Oxford, Oxford, United Kingdom; ^4^Department of Clinical and Movement Neurosciences, UCL Queen Square Institute of Neurology, London, United Kingdom

**Keywords:** unimanual motor learning, bimanual motor learning, transcranial alternating current stimulation, bihemispheric stimulation, phase synchrony, beta activity

## Abstract

Many tasks require the skilled interaction of both hands, such as eating with knife and fork or keyboard typing. However, our understanding of the behavioural and neurophysiological mechanisms underpinning bimanual motor learning is still sparse. Here, we aimed to address this by first characterising learning-related changes of different levels of bimanual interaction and second investigating how beta tACS modulates these learning-related changes. To explore early bimanual motor learning, we designed a novel bimanual motor learning task. In the task, a force grip device held in each hand (controlling *x*- and *y*-axis separately) was used to move a cursor along a path of streets at different angles (0°, 22.5°, 45°, 67.5°, and 90°). Each street corresponded to specific force ratios between hands, which resulted in different levels of hand interaction, i.e., unimanual (*Uni*, i.e., 0°, 90°), bimanual with equal force (*Bi*_*eq*_, 45°), and bimanual with unequal force (*Bi*_*uneq*_ 22.5°, 67.5°). In experiment 1, 40 healthy participants performed the task for 45 min with a minimum of 100 trials. We found that the novel task induced improvements in movement time and error, with no trade-off between movement time and error, and with distinct patterns for the three levels of bimanual interaction. In experiment 2, we performed a between-subjects, double-blind study in 54 healthy participants to explore the effect of phase synchrony between both sensorimotor cortices using tACS at the individual’s beta peak frequency. The individual’s beta peak frequency was quantified using electroencephalography. 20 min of 2 mA peak-to-peak amplitude tACS was applied during task performance (40 min). Participants either received in-phase (0° phase shift), out-of-phase (90° phase shift), or sham (3 s of stimulation) tACS. We replicated the behavioural results of experiment 1, however, beta tACS did not modulate motor learning. Overall, the novel bimanual motor task allows to characterise bimanual motor learning with different levels of bimanual interaction. This should pave the way for future neuroimaging studies to further investigate the underlying mechanism of bimanual motor learning.

## Introduction

In everyday life, we perform countless movements with our hands. Some, such as writing, drawing, or eating with chopsticks, require one hand alone, whereas others require the skilled interaction of both hands. For bimanual movements, the two hands either perform actions in a *similar* manner, i.e., with equal contributions between hands like opening a drawer or rope skipping, or actions in a *different* manner, i.e., with unequal contributions between hands like eating with knife and fork or keyboard typing.

Although many daily life tasks require skilled bimanual interactions, our understanding of their behavioural and neurophysiological underpinnings is still sparse (Kelso et al., 1979; Swinnen, 2002; Swinnen and Gooijers, 2015). Bimanual interactions are mainly studied using simple finger tapping, sequence tapping and the simultaneous or alternating flexion/extension of individual fingers (Shammi et al., 1998; Bangert et al., 2010; Takeuchi et al., 2012; Sallard et al., 2014; Koppelmans et al., 2015; Loehrer et al., 2016; Kajal et al., 2017). These studies have advanced our understanding of which regions are involved in bimanual interaction and how they communicate with each other (see for review Swinnen, 2002; Swinnen and Gooijers, 2015), but the largely artificial tasks used remain distant to daily life and often do not require learning.

The few existing studies examining complex bimanual interactions that require learning have investigated rhythmical/cycling movements in a bimanual tracking task using either rotating levers (Kelso et al., 1986; Swinnen, 1998; Sisti et al., 2011) or devices like crank handles (Preilowski, 1972; Fagard et al., 1985; Mueller et al., 2009) to draw on a screen. These studies found condition-specific improvements in performance following extensive and repetitive training, with faster and more accurate performance in conditions requiring equal contributions between hands in comparison to conditions requiring unequal contributions (Fagard et al., 1985; Mueller et al., 2009). While these studies provided essential insights into the temporal aspects of rhythmical/cycling bimanual movement, most daily life bimanual movements are not rhythmic or cyclic by nature. Many bimanual movements require a distinct yet unique cooperative contribution of each hand, such as bottle opening or eating with knife and fork. Cooperative bimanual interactions and their learning are highly understudied (see for a review Obhi, 2004). Some attempts have been made to develop task setups to investigate goal-directed and cooperative interactions, e.g., in which one hand stabilises the other (Dietz et al., 2015) or in which a cursor had to be navigated across a complex circuit (Doost et al., 2017). However, those studies focused on bimanual interactions requiring equal contributions of hands and did not compare these to unequal contributions between hands. Investigating both equal and unequal contributions of hands will allow a richer description of bimanual movements and their learning and will provide new insights into how the brain coordinates cooperative bimanual interactions.

According to the communication through coherence hypothesis, neural oscillations in multiple brain regions align in frequency and phase (Fries, 2005, 2015). This alignment in frequency and phase, also known as coherence or synchrony, has been proposed to be a central mechanism for inter-regional communication (Fries, 2005, 2015). For movements, many studies have demonstrated the importance of neural activity in the beta band (15–30 Hz) for neural communication, as neurons desynchronise during movement preparation and execution (event-related desynchronisations, ERD) and synchronise post- movement (event related synchronisations, ERS; Pfurtscheller and da Silva, 1999; Neuper and Pfurtscheller, 2001). In complex bimanual motor learning, the role of beta dynamics is not completely understood (see Gerloff and Andres, 2002 for a review). Some evidence from finger tapping, rotation, or flexion and extension, suggests that interhemispheric coherence between left and right M1 in the beta band is present during bimanual motor learning (Gerloff et al., 1998; Serrien and Brown, 2002; Kajal et al., 2017) and increases with coordinative effort and task demand (Andres et al., 1999; Loehrer et al., 2016). Further, beta dynamics are different in movements requiring equal and unequal contributions of hands, whereby equal contributions between hands present lower interhemispheric coherence (Boonstra et al., 2007; Serrien, 2008). This difference is thought to imply that equal contributions between hands require lower effort (Serrien, 2008), whereas unequal contributions between hands require more effort indicated by increased neural communication (Serrien and Brown, 2002; Serrien et al., 2003). It is also likely that beta activity plays a causal part in learning: the ERD become more pronounced with unimanual motor learning and the ERS with motor adaptation (Tan et al., 2014; Zich et al., 2018).

However, the evidence discussed above does not test the role of hemispheric synchrony of beta activity in complex bimanual motor learning. We therefore wanted to test if frequency and phase alignment between hemispheres improves bimanual motor learning. Specifically, we wanted to test the effects of beta phase synchrony compared to phase desynchrony between hemispheres on learning equal and unequal bimanual movements. This allows to directly test the role of hemispheric synchrony of beta activity in bimanual motor learning and thereby give insights into the link between oscillatory activity and behaviour.

Previous studies have used transcranial alternating current stimulation (tACS), a non-invasive brain stimulation approach, applying a weak sinusoidal current to one or more brain areas (see for a review Antal and Paulus, 2013) to entrain cortical network coherence (see for a review Herrmann et al., 2013 or Antal and Herrmann, 2016). Specifically, the application of tACS is thought to shift the ongoing activity in the brain toward the frequency and phase alignment of the stimulation and thereby change the coherence between brain areas (Antal et al., 2008; Ali et al., 2013; Reato et al., 2013; Weinrich et al., 2017). Thus, tACS provides a tool for investigating the causal role of neural oscillations for behaviour (for a review see Riddle and Frohlich, 2021).

Given the central role of beta dynamics, it is perhaps not surprising that driving brain activity toward the natural individual beta activity during movements influences motor performance when applied either to one (Pogosyan et al., 2009; Joundi et al., 2012; Wach et al., 2013) or both sensorimotor cortices (Heise et al., 2019). Further, evidence suggests that the effects of tACS are most pronounced when the stimulation frequency matches the natural frequency in the brain (Zaehle et al., 2010; Thut et al., 2011; Ali et al., 2013; Reato et al., 2013; Schilberg et al., 2018). In addition, there is some evidence that shifting the phase of tACS is behaviourally relevant when stimulation was applied during unimanual motor learning on one hemisphere (Polanıa et al., 2012; Violante et al., 2017). Phase synchrony between hemispheres was investigated by Heise et al. (2019), who could show that in-phase stimulation, i.e., phase synchrony between hemispheres, at 10 Hz and 20 Hz benefited task switching during bimanual tapping. Helfrich et al. (2014a) could demonstrate that interhemispheric functional coherence was modulated in a phase-specific way when bilateral high-density tACS at 40 Hz was applied with simultaneous electroencephalographic (EEG) recordings. Similar phase-specific effects were shown by Schwab et al. (2019) who applied bihemispheric high-definition alpha tACS. Thereby, modifying the phase of beta tACS by stimulating between hemispheres, seems to be promising for modulating learning-related changes in behaviour.

In experiment 1, we aimed to characterise the learning of different levels of bimanual interaction using a novel task. The task was designed to capture unimanual learning as well as different levels of bimanual learning to study bimanual cooperation and directly compare equal and unequal contribution of hands. Our task was inspired by Doost et al. (2017), who used a bimanual version of a circuit game from Lefebvre et al. (2012) to study bimanual motor learning with equal contribution of hands. The task involved moving a cursor along an angled street, where the two hands controlled orthogonal axes. To compare different levels of bimanual interaction, our task consisted of streets requiring a unimanual response, streets where both hands were required equally (bimanual equal) and streets where both hands were required at different levels (bimanual unequal). We hypothesised that performance would differ between the three conditions (main effect), that learning-related changes would be evident (main effect), and finally that the learning-related changes would differ between the three conditions (interaction). Further, we explored whether the bimanual skill acquired in the task is specific to the task or whether it transferred to a transposition of the axes.

In experiment 2, we investigated test the role of hemispheric synchrony of beta activity while learning the task with in-phase (phase synchrony between hemispheres) and out-of-phase (phase desynchrony between hemispheres) tACS at the individual beta-peak frequency above both sensorimotor cortices. We hypothesised that beta tACS modulates learning-related changes. As previous studies found different beta dynamics for equal and unequal contributions between hands, i.e., less beta coherence between hemispheres in equal compared to unequal contributions, we expected in-phase stimulation to improve learning in the bimanual equal condition and out-of-phase stimulation to improve learning in the bimanual unequal condition.

## Materials and Methods

### Bimanual Motor Learning Task

**FIGURE 1:**
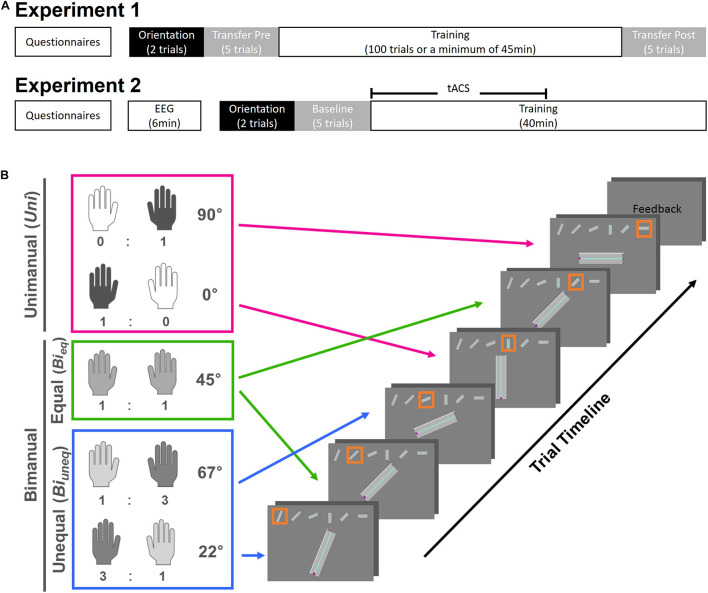
Schematic overview of the experimental timeline **(A)** and task design **(B)**. **(A)** In experiment, participants filled in questionnaires and performed 45 min of the training task with a minimum of 100 trials. In experiment 2, after filling in questionnaires and identifying the beta peak frequency with EEG, participants performed the training task for 40 min. For each experiment, before learning the training task, two orientation trials were given and the hand-axis configuration was swapped, i.e., transposed, for five trials (Transfer Pre). In experiment 1, this transposition was also performed for five trials after learning (Transfer Post). In experiment 2, the transposition was used as a baseline measurement to allocate participants to stimulation groups. **(B)** Each trial consisted of three conditions, i.e., *Uni* (pink), *Bi*_*eq*_ (green), and *Bi*_*uneq*_ (blue). The ideal left- and right-hand ratios with the corresponding street angles are shown. Each condition was present twice in one trial; therefore, one trial would consist of one path with six streets. Each street was displayed in the middle of the screen with the whole path on top. The orange frame indicated on which street participants were currently on. After finishing the path, participants received feedback on their performance (time and accuracy) and started the next path in a self-paced manner.

We designed a novel bimanual motor learning task to characterise the learning of a complex, multi-level motor task with different levels of bimanual interaction. The task was implemented as a racing game as context-dependent effects in motor control have been shown to be important (Steinberg and Vogt, 2015). In the task, a cursor had to be moved through a path consisting of six streets (see Figure 1B). Participants were instructed to move the cursor as quickly and accurately as possible and that both were equally important. The cursor movements were controlled by two force grippers (one controlling the horizontal movement of the cursor and one controlling the vertical movement of the cursor). Which hand controlled vertical/horizontal movement was balanced across the group.

#### Different Levels of Bimanual Interaction

Each path (i.e., trial) contained six streets that were angled to produce different ideal force ratios from the two hands (see Figure 1B). Specifically, only one hand was needed to navigate the cursor on the vertical (0°) and horizontal (90°) street in the **unimanual condition (*Uni*)**. These streets corresponded to an ideal force-ratio of 1:0 or 0:1. The **bimanual equal condition (*Bi*_***eq***_)** required an ideal force-ratio of 1:1 between the two hands to move the cursor on streets angled at 45°. Finally, the **bimanual unequal condition (*Bi*_***uneq***_)** required an unequal force contribution between hands to produce an ideal force ratio of 3:1 or 1:3, to move the cursor on streets angled at either 22.5° or 67.5°. Each path contained two streets from each condition, pseudo-randomised to avoid memorisation of the path, and such that two unimanual conditions or two bimanual equal conditions did not occur successively.

#### Trial Structure

The whole path was displayed at the top of the screen throughout the trial (see Figure 1B). The current street was highlighted by an orange frame and displayed in the middle of the screen. At the beginning of each street the cursor was at the starting position (i.e., in the middle of the width of the path, at the bottom-left position). If the cursor hit either side of the street, the cursor was reset to the starting position. When the cursor reached the end of the street (defined as last 5% of the whole length of the street) the next street started immediately. At the end of the whole trial (i.e., six streets), feedback on movement time and accuracy of that trial was shown. The inter-trial interval was self-paced.

#### Response Devices

Data was acquired with the Grip Force Bimanual Fiber Optic Response Pad (HHSC-2x1-GRFC-V2, Current Designs Inc., Philadelphia, PA, United States) connected to an electronic interface box (932, Current Designs Inc., Philadelphia, PA, United States). The task was programmed in MATLAB [version 9.5.0.944444 (R2018b), The MathWorks Inc., Natick, MA, United States], using the Psychophysics Toolbox (version 3.0.14) extensions (Brainard, 1997; Pelli, 1997; Kleiner et al., 2007).

The force grippers were calibrated to the participant’s maximum force by squeezing the grippers five times with maximum force. Each gripper was calibrated individually and specifically to the hand holding the gripper (grippers were not swapped). Cursor movement required a minimum of 15% of the participant’s maximum force. If participants applied 100% of their maximum force, the cursor would move 25 pixels per refresh rate of the monitor (60 times per second). After the cursor was moved to the end of the street, participants had to reduce the force to less than 2% of their maximum force for the next street to be displayed.

#### Dependent Variables

Movement time and error were calculated for each path and each street. Movement time was defined as the time from movement onset to offset of the cursor. To calculate the trial-level movement time across the whole path, the time from movement onset to offset of the cursor of all six streets were summed. Error was obtained for each timepoint (sampling rate = 60 Hz) by calculating the distance of the cursor from the ideal line (midline of the streets) using Heron’s Formula. The error for one street constitutes the root mean square of the error per timepoint. The error for the whole path (trial-level error) was defined as the sum of the errors of all six streets. In addition, the number of times the cursor was reset to the starting position in each street, was measured independently as the reset index. However, for this data analysis the reset index was captured by the movement time, i.e., resets increase movement time.

Online trial-level movement time and error were fed back to the subject after each path. In addition, movement time and error were calculated offline for each of the three conditions (*Uni*, *Bi*_*eq*_, and *Bi*_*uneq*_) separately.

### Experiment 1

#### Participants

Forty healthy participants (24 females, mean age = 25.3 years, SD = 3.55 years) gave their written informed consent to participate in the study in accordance with Central University Research Ethics Committee approval (University of Oxford; MSD-IDREC-R61309/RE001) and in accordance with the Declaration of Helsinki. The number of participants was chosen based on experience and on previous studies investigating bimanual motor learning (Doost et al., 2017). Participants received monetary compensation for taking part in the study.

Participants reported no history of neurological or psychiatric disorders. All had normal or corrected-to-normal vision and were right-handed, as assessed with the 10 item Edinburgh Handedness Inventory (Oldfield, 1971).

#### Experimental Design

The experiment consisted of one session (see Figure 1A). First, participants completed questionnaires regarding the bimanual competence and an Achievement Motivation Inventory (AMI). After calibration, two orientation trials for familiarisation were completed, followed by five trials of the transfer task (Transfer Pre). Next, the training task was practiced for a minimum of 45 min or 100 trials. The time on task, i.e., 45 min, and number of repetitions, i.e., 100 trials, was based on extensive piloting which suggested that these criteria were sufficient for learning the task. After the training task, five trials of the transfer task were performed (Transfer Post). The transfer task was identical to the training task, with the only exception that the hand-axis configuration was swapped to assess the effect of transposition.

#### Questionnaires

In the absence of available standardised questionnaires, we designed a short questionnaire to estimate bimanual competence. This included a self-report about a range of bimanual activities including sports, hobbies, keyboard and phone typing, musical instruments, and video games, and was scored overall from 1 (basic) to 3 (expert). Additionally, participants completed the short version of the AMI. The AMI is a psychological test inventory with 30 questions measuring different aspects of work-related achievement motivation. The test items were translated from German «Leistungsmotivationsinventar (LMI)» (Schuler and Prochaska, 2001).

#### Statistical Analysis

To investigate learning-related changes, the first and last ten trials of the training task were compared both on the “trial level” and on the “condition level” with error and movement time as dependent variables. To investigate the trade-off between changes in movement-time and error (i.e., speed-accuracy) and to investigate the difference in learning between conditions, change scores (i.e., difference between first and last ten trials of the training task) were used.

Data were analysed with paired samples *t*-tests and repeated-measures analysis of variance (ANOVA). Significant interactions were followed up using paired samples *t*-tests. Furthermore, correlations were assessed using the Pearson’s correlation.

All reported *p*-values for *t*-tests are two-tailed and the significance level was set at *p* < 0.05 (Bonferroni corrected when appropriate). Statistical analysis was performed using the open-source software JASP (version 0.9.2, JASP Team, 2019).

### Experiment 2

#### Participants

An additional sample (independent sample from experiment 1) of 55 right-handed healthy individuals took part in this study in accordance with Central University Research Ethics Committee approval (University of Oxford; MSD-IDREC-R65118/RE001) and in accordance with the Declaration of Helsinki. In the absence of previous studies on phase-specific beta tACS effects across hemispheres, no effect sizes were available for a power calculation. Participants were monetarily compensated for their participation. They reported no neurological or psychiatric disorders, or contra-indications for brain stimulation and had normal or corrected to normal vision. Right-handedness was assessed by the shortened (10 item) Edinburgh Handedness Inventory (Oldfield, 1971). One participant was excluded from the experiment because no beta peak frequency was detectable. The final sample comprised 54 individuals (28 females, mean age = 24.05 years, SD = 4.76 years).

#### Experimental Design

**FIGURE 2:**
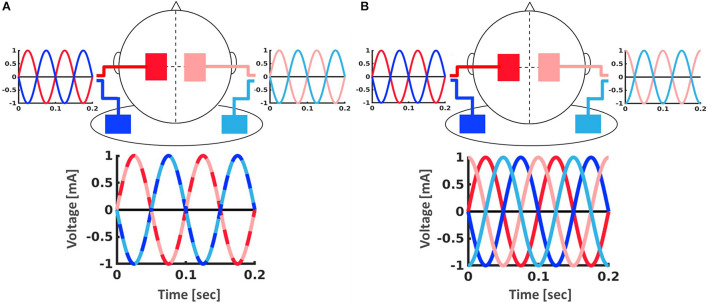
Setup for the tACS application with two stimulators (dark and light) which were each connected to one electrode over M1 (red) and one electrode on the ipsilateral shoulder (blue). **(A)** In-phase stimulation (0° phase shift between hemispheres). **(B)** Out-of-phase stimulation (90° phase shift between hemispheres).

To investigate the effect of phase synchrony between hemispheres on bimanual motor learning, we conducted a double-blind sham-controlled study with three groups (in-phase, out-of-phase, sham) differing in the relative phases of the applied sinusoidal current between the motor cortices (see Figure 2).

Questionnaires assessing bimanual competence and achievement motivation were completed in the beginning of the session as described in section “Materials and Methods: Experiment 1.” Next, EEG was recorded while participants performed simple hand squeezes, to identify the participant’s beta peak frequency, which was then used as the tACS stimulation frequency.

Participants then performed the bimanual task as described in section “Materials and Methods: Experiment 1.” Participants started with two orientation trials for familiarisation, followed by five trials (baseline measurement) and 40 min of the training task (see Figure 1A). The time on task, i.e., 40 min, was based on the findings of experiment 1. To avoid carry-over effects from the baseline measurement to the training task, the hand-axis configuration was swapped for the baseline measurement, like in the transfer task in experiment 1. During the first 20 min of the training task, participants received tACS at their individual beta-peak frequency. At the end of the session, participants were asked to report their guess of stimulation group (sham/active) and how certain they were on a scale from 1 to 7.

#### Electroencephalography Acquisition and Analysis

To define the individual’s beta peak frequency, we recorded 6 min of EEG while participants performed a simple motor task. Participants held one squeeze ball in each hand and were asked to fixate on a cross displayed on a screen. An arrow was presented for 200 ms, which pointed either left or right, in response to which participants squeezed the corresponding hand. Participants were instructed to perform one hand muscle contraction with maximum force and then immediately relax while otherwise being as still as possible. This was practiced before the beginning of the EEG recording and visually monitored by the experimenter. The order of left and right squeezes was pseudo-randomised. Stimuli for the EEG recording were presented with OpenViBE Designer (version 2.2.0, Renard et al., 2010).

EEG data were recorded with a modified Emotiv^1^ system as reported previously (Debener et al., 2012; de Vos et al., 2014; Zich et al., 2015). Briefly, the original hardware (128 Hz sampling rate; 0.16 and 45 Hz bandpass) was relocated into a small and light box (49 mm × 44 mm × 25 mm; 48 g total weight), which was attached to an infra-cerebral electrode cap from Easycap^2^ in a small pocket at the back of the head. The EEG data were collected from sintered Ag/AgCl electrodes positioned at the 10–20 sites Cp5, Cp3, C3, C4, Cp4, Cp6 with a central frontopolar electrode (Fz) as reference and a central parietal (Pz) as ground. Data was acquired with OpenViBE Acquisition Server (version 2.2.0, Renard et al., 2010).

EEG data were analysed using MATLAB (The MathWorks Inc., Natick, MA, United States) and EEGLAB14_1_1b (Delorme and Makeig, 2004). EEG data were high-pass filtered (5 Hz, finite impulse response, filter order 212) and subsequently low-pass filtered (40 Hz, finite impulse response, filter order 44). Data were segmented from −2 s to 4 s (relative to the trigger indicating movement onset) and baseline corrected (−1.5 s to −0.5 s). Next, segments containing residual artefacts were rejected (EEGLAB functions pop_jointprob.m, pop_rejkurt.m, both SD = 3). A time-frequency analysis (Morlet wavelet transform, 1 Hz frequency steps) was performed on these segments for each of the six channels. The frequency with the strongest movement-induced event-related desynchronisation was selected as stimulation frequency.

#### Group Allocation and Beta Transcranial Alternating Current Stimulation Application

Participants were stratified and pseudo-randomised to ensure comparability between groups in terms of beta peak frequency, baseline performance of movement time and error, as well as scores of bimanual competence and achievement motivation, to minimise variance across groups (Sella et al., 2021). Group allocation was double-blind, i.e., participant and experimenter did not know about the stimulation group the participant was allocated to. A third person delivered the stimulation.

Participants received either in-phase, i.e., 0° phase shift between hemispheres, (20 min), out-of-phase, i.e., 90° phase shift between hemispheres, (20 min) or sham (3 s of stimulation) tACS with a ramp up and down for 10 s. Two bipolar DC Plus stimulators (NeuroConn, Ilmenau, Germany) were each connected to a pair of conductive rubber electrodes (5 cm × 7 cm), with one electrode over M1 (C3/C4) and one electrode on the ipsilateral shoulder (Figure 2). High-chloride EEG electrode paste was used as a conducting medium and impedance was kept below 5 Ω at all times. Stimulation was applied at 2 mA peak-to-peak amplitude during the first 20 min of performance of the bimanual motor learning task (Figure 1A).

#### Statistical Analysis

Analyses were performed as in experiment 1. In addition, groups were compared regarding individual’s beta peak frequency, pre-task movement time and error, as well as questionnaires regarding the AMI and bimanual competence using Bayesian inference. Bayesian inference was used as no differences were expected. The Bayes factors (BFs) is the ratio of the likelihood of one particular hypothesis to the likelihood of another. We categorised BFs based using the heuristic classification scheme (Lee and Wagenmakers, 2013, p. 105; adjusted from Jeffreys, 1961). Bayesian inference was performed using Bayesian inference (JASP, JASP Team, 2019, version 0.9.2) with default priors.

Blinding to tACS condition was evaluated using the index introduced by Bang et al. (2004) once for the sham group and once for the two stimulation groups (out-of-phase, in phase) together. If the participant were 50% or less certain, this was coded as a “don’t know” for the blinding indices (BI) calculation.

In addition, we explored whether accounting for baseline performance influenced the results. We used adjustment methods of calculating a performance change between baseline and training task (i.e., change score), as previously suggested by Vickers and Altman (2001) and Zhang et al. (2014). To calculate the change score, we subtracted the baseline from the first ten trials and last ten trials before the end of stimulation, both on the trial and condition level once for movement time and once for error.

## Results

### Experiment 1

The aim of **experiment 1** was to validate the novel motor task by characterising learning-related changes at different levels of bimanual interaction. Questionnaire outcome measures of bimanual competence (mean = 2.075, SD = 0.829, range: 1–3) and AMI (mean = 6.025; SD = 1.609, range: 2–9) did not interact with learning the task (all *p*’s > 0.05).

#### Learning of the Task Reduced Both Movement Time and Error

**FIGURE 3:**
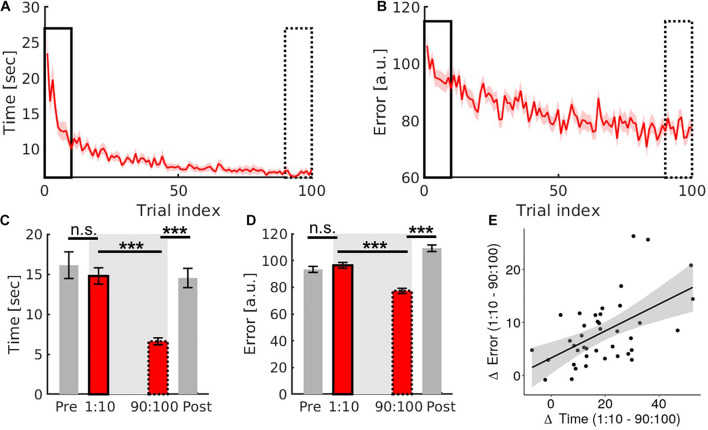
Results of the training task (red) and transfer task (grey). **(A,B)** Qualitative presentation of the reduction of movement time and error with learning. **(C,D)** Quantitative comparison of the first ten (solid frame) and the last ten trials (dashed frame) of the training task with the transfer task conducted before (pre) and after (post) the training task. **(E)** Correlation plot of movement time changes and error changes in the training task. Error bars are standard errors of the mean. Significance stars ^∗∗∗^ mean *p* < 0.001.

Learning reduced movement time and error on the trial level (Figures 3A,B). Two paired samples *t*-tests (first, last trials) were conducted. We observed a significant reduction in movement time (*t*_1,39_ = 8.28, *p* < 0.001, Cohen’s *d* = 1.31) and error (*t*_1,39_ = 9.03, *p* < 0.001, Cohen’s *d* = 1.43) (see Figures 3C,D).

To investigate whether these behavioural improvements were mediated by a trade-off between changes in movement-time and error (i.e., speed-accuracy trade-off), a Pearson’s correlation was performed. There was a significant positive correlation between the difference between the first and last ten trials of movement time and error (*r* = 0.55, *p* = 0.001, *CI* = [0.73, 0.28]), suggesting that there was no speed-accuracy trade-off (see Figure 3E).

#### Behavioural Gains Are Specific to the Trained Hand Configuration

We then investigated if the learning-related improvements were specific or whether they generalised to the transfer task (Figure 1). Two 2 × 2 ANOVAs with within-subject factors of task type (training and transfer) and timepoint (before and after learning), were conducted for movement time and error separately. We found significant task × timepoint interactions for both learning metrics (movement time: *F*_1,39_ = 26.003, *p* < 0.001, *n^2^_*p*_* = 0.4; error: *F*_1,39_ = 107.683, *p* < 0.001, *n^2^_*p*_* = 0.734, Figures 3C,D). See Supplementary Table 1 for main effects.

*Post hoc* tests (Bonferroni corrected) revealed that performance during the pre-learning transfer task and the first ten trials of the training task was comparable (movement time: *t*_1,39_ = −0.931, *p* = 0.358, Cohen’s *d* = −0.147; error: *t*_1,39_ = 1.116, *p* = 0.271, Cohen’s *d* = 0.177). However, performance during the post-learning transfer task was significantly worse than during the last ten trials of the training task (movement time: *t*_1,39_ = −7.375, *p* < 0.001, Cohen’s *d* = −1.166; error: *t*_1,39_ = −12.462, *p* < 0.001, Cohen’s *d* = −1.97, see Figures 3C,D). This suggests that learning-related changes do not transfer to this transfer task.

#### Distinct Learning Patterns for the Different Levels of Bimanual Interaction

**FIGURE 4:**
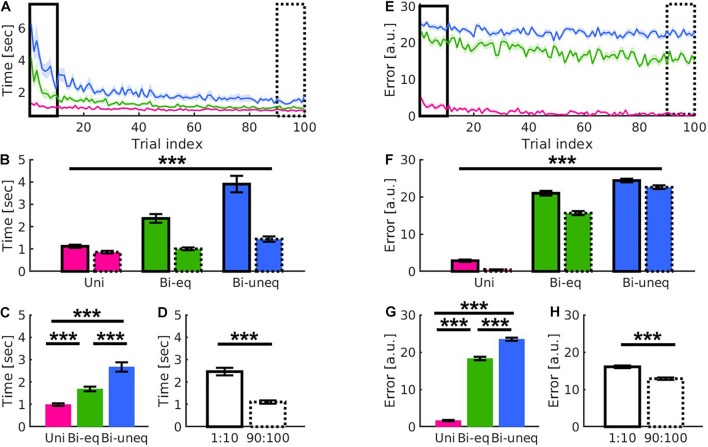
Results showed distinct patterns for the different levels of bimanual interaction (*Uni*: pink; *Bi*_*eq*_: green; *Bi*_*uneq*_: blue) in movement time **(A–D)** and error **(E–H)**. **(A,E)** Qualitative presentation of the interaction of condition and time. **(B,F)** Quantitative presentation of the interaction of condition and time by comparing first ten (solid frame) and last ten (dashed frame) trials. This suggests that movement time and error decreased differently for the three conditions over time. **(C,G)** Quantitative presentation of the main effect of condition. **(D,H)** Quantitative presentation of the main effect of time. Error bars are standard errors of the mean. Significance stars ^∗∗∗^ mean *p* < 0.001.

Each trial consisted of six streets, of which two required unimanual (*Uni*) force, two bimanual with equal force (*Bi*_*eq*_), and two bimanual with unequal force (*Bi*_*uneq*_) (see Figure 1). The three conditions differed during learning (Figures 4A,E). To investigate any differences in learning-related changes across the three conditions, we performed repeated-measures 2 × 3 ANOVAs with within-subject factors of time (first, last trials) and condition (*Uni, Bi_*eq*_, Bi_*uneq*_*) for movement time and error. For both behavioural metrics there was a significant main effect of time (movement time: *F*_1,39_ = 68.61, *p* < 0.001, *n^2^_*p*_* = 0.638; error: *F*_1,39_ = 81.55, *p* < 0.001, *n^2^_*p*_* = 0.676; Figures 4D,H), condition (movement time: *F*_2,78_ = 52.53, *p* < 0.001, *n^2^_*p*_* = 0.574; error: *F*_2,78_ = 1460.82, *p* < 0.001, *n^2^_*p*_* = 0.974; Figures 4C,G), and a significant time × condition interaction (movement time: *F*_2,78_ = 27.13, *p* < 0.001, *n^2^_*p*_* = 0.41; error: *F*_2,78_ = 13.44, *p* < 0.001, *n^2^_*p*_* = 0.256; Figures 4B,F).

The main effect of condition was followed up using paired samples *t*-tests (Bonferroni corrected). All comparisons were significant (all *p*’s < 0.001; Figures 4C,G). To follow up the time × condition interaction, we compared the amount of learning between the three conditions using change scores, i.e., difference between first and last ten trials (Bonferroni corrected). For movement time, the amount of learning was significantly larger for *Bi*_*uneq*_ compared to *Bi*_*eq*_ (*p* < 0.003) and *Uni* (*p* < 0.001), whereby the amount of learning was also significantly larger for *Bi*_*eq*_ when compared with *Uni* (*p* < 0.001). For error, the amount of learning was significantly larger for *Bi*_*eq*_ compared to *Bi*_*uneq*_ (*p* < 0.001) and *Uni* (*p* < 0.001), whereby the amount of learning did not differ between *Uni* and *Bi*_*uneq*_ (*p* = 0.26).

### Experiment 2

The aim of **experiment 2** was to investigate the effects of beta tACS on learning-related changes in movement time and error. Similar to experiment 1, questionnaire outcome measures of bimanual competence and AMI did not interact with learning (all *p*’s > 0.05), but were comparable to those of experiment 1, see section “Results: Experiment 1.”

#### No Baseline Differences Between Stimulation Groups

We tested whether groups were comparable at baseline measures (beta peak frequency, pre-task movement time and error, questionnaires). We conducted five 1 × 3 Bayesian ANOVAs, one for each group allocation parameter (Table 1). The results support the null hypothesis of no differences between the groups at baseline.

**TABLE 1:** No baseline differences between groups for the group allocation parameters (mean ± SD and range).

	**Out-of-phase**	**In-phase**	**Sham**	**Stats**
Beta Peak (Hz)	19.39 ± 3.33 (14–26)	19.39 ± 2.35 (16–26)	19.61 ± 1.61 (16–22)	BF_10_ = 0.150
Error (a.u.)	16.13 ± 2.38 (11.38–19.62)	16.51 ± 2.78 (11.82–23.35)	16.53 ± 1.87 (12.7–19.48)	BF_10_ = 0.162
Time (s)	4.24 ± 1.49 (2.35–7.12)	4.14 ± 1.96 (2.06–9.67)	4.07 ± 1.96 (1.85–9.3)	BF_10_ = 0.149
AMI	5.33 ± 1.61 (3–8)	5.72 ± 2.24 (1–9)	5.67 ± 1.5 (3–8)	BF_10_ = 0.173
Bim. competence	2.17 ± 0.86 (1–3)	2.06 ± 0.8 (1–3)	1.78 ± 0.81 (1–3)	BF_10_ = 0.313

#### Blinding Successful for Sham, but Not for Active Groups

Success of blinding was quantified using the Bang Index (BI). For the tACS groups (both in-phase and out-of-phase together), the BI was 0.72 (*CI* = [0.58–0.87]) and for the sham group −0.056 (*CI* = [−0.38 to 0.27]), suggesting that blinding was successful for the sham group, but not for the active groups.

#### Transcranial Alternating Current Stimulation Does Not Modulate Learning

To test whether beta tACS affected motor learning, we conducted two 2 × 3 ANOVAs with a within-subject factor of time [first, last (before the end of stimulation) trials] and between-subject factor of group (in-phase, out-of-phase, sham) on the trial level. Similarly, to experiment 1, there was a significant main effect of time for both metrics (movement time: *F*_1,51_ = 39.42, *p* < 0.001, *n^2^_*p*_* = 0.436; error: *F*_1,51_ = 30.62, *p* < 0.001, *n^2^_*p*_* = 0.375). However, there was no significant main effect of group (movement time: *F*_2,51_ = 0.369, *p* = 0.693, *n^2^_*p*_* = 0.014; error: *F*_2,51_ = 0.561, *p* = 0.574, *n^2^_*p*_* = 0.022), nor a significant time × group interaction (movement time: *F*_2,51_ = 2.1, *p* = 0.133, *n^2^_*p*_* = 0.076; error: *F*_2,51_ = 0.403, *p* = 0.67, *n^2^_*p*_* = 0.016). These results suggest that individuals successfully learned the task, but that beta tACS did not modulate the learning.

**FIGURE 5:**
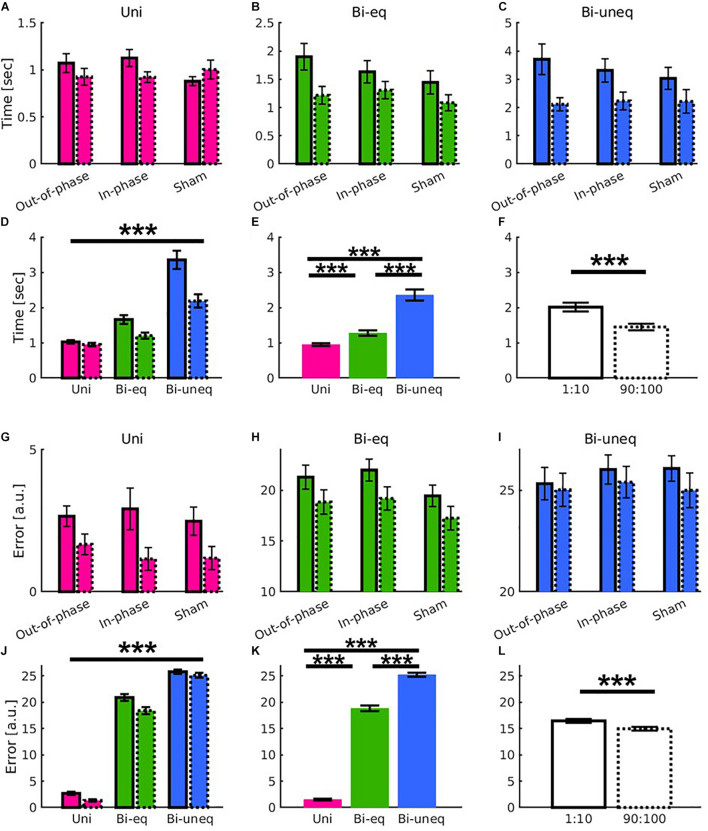
Results showed learning yields main effects of time and condition as well as distinct learning-related patterns for the different conditions (*Uni*: pink; *Bi*_*eq*_: green; *Bi*_*uneq*_: blue), but beta tACS did not modulate the patterns in either movement time **(A–F)** or error **(G–L)** as shown in the comparison of the first ten (solid frame) and last ten trials before the end of stimulation (dashed frame). **(A–C,G–I)** Interaction between time × condition × group individually scaled for greater visibility. **(D,J)** Significant interaction between time × condition. **(E,K)** Main effect of condition. **(F,L)** Main effect of time. Error bars are standard errors of the mean. Significance stars ^∗∗∗^ mean *p* < 0.001.

To investigate if beta tACS had an influence on the different levels of bimanual interaction, we performed two 2 × 3 × 3 ANOVAs with within-subject factors of time [first, last (trials before the end of stimulation)] and condition (*Uni, Bi_*eq*_, Bi_*uneq*_*) and a between-subject factor of group (in-phase, out-of-phase, sham). For both metrics, there was a significant main effect of time (movement time: *F*_1,51_ = 39.85, *p* < 0.001, *n^2^_*p*_* = 0.439; error: *F*_1,51_ = 33.05, *p* < 0.001, *n^2^_*p*_* = 0.393; Figures 5F,L), condition (movement time: *F*_2,102_ = 79.151, *p* < 0.001, *n^2^_*p*_* = 0.608; error: *F*_2,102_ = 1330.206, *p* < 0.001, *n^2^_*p*_* = 0.963; Figures 5E,K) and a significant time × condition interaction (movement time: *F*_2,102_ = 20.52, *p* < 0.001, *n^2^_*p*_* = 0.287; error: *F*_2,102_ = 4.77, *p* = 0.011, *n^2^_*p*_* = 0.085; Figures 5D,J). However, there were no significant effects of group (all *p*’s > 0.1; see Figures 5A–C,G–I and Table 2).

**TABLE 2:** Results of the 2 × 3 × 3 ANOVA.

		Movement time			Error		
			
	df	*F*	*p*	*n^2^_*p*_*	*F*	*p*	*n^2^_*p*_*
Time	1,51	39.85	<0.001	0.439	33.05	<0.001	0.393
Condition	2,102	79.151	<0.001	0.608	1330.206	<0.001	0.963
Group	2,51	0.38	0.686	0.015	0.57	0.569	0.022
Time × Condition	2,102	20.52	<0.001	0.287	4.765	0.011	0.085
Time × Group	2,51	2.194	0.122	0.079	0.285	0.753	0.011
Condition × Group	4,102	0.148	0.964	0.006	1.322	0.267	0.049
Time × Condition × Group	4,102	0.762	0.552	0.029	0.206	0.935	0.008

The main effect of condition was followed up using paired samples *t*-tests (Bonferroni corrected). All comparisons were significant conditions (all *p*’s < 0.001; Figures 5E,K). As in experiment 1, we followed up the time × condition interaction using change scores (Bonferroni corrected). For movement time, the amount of learning was significantly larger for *Bi*_*uneq*_ compared to *Bi*_*eq*_ and *Uni*, whereby the amount of learning was also significantly larger for *Bi*_*eq*_ when compared with *Uni* (all *p*’s < 0.001). For error, the amount of learning was significantly larger for *Bi*_*eq*_ compared to *Bi*_*uneq*_ (*p* = 0.011), whereby the amount of learning did not differ between *Uni* and *Bi*_*eq*_ (*p* = 0.076), and *Uni* and *Bi*_*uneq*_ (*p* = 0.089).

Given the lack of blinding during active beta tACS, we specifically tested for a double dissociation of out-of-phase, in-phase tACS and *Bi_*eq*_, Bi_*uneq*_* condition. Therefore, two 2 × 2 × 2 ANOVAs with within-subject factors of time [first, last (before the end of stimulation) trials] and condition (*Bi_*eq*_, Bi_*uneq*_*) and a between-subject factor of group (in-phase, out-of-phase). Results revealed no significant main effect of group or interaction with group (all *p*’s > 0.1).

Finally, to ensure that any differences in baseline did not affect the results, we explored whether adjusting for pre-task performance influenced the results. Accounting for baseline measures by calculating the change between baseline performance and training had no influence on the group effects.

## Discussion

We designed a novel motor learning task to characterise bimanual motor learning with different levels of bimanual interaction (experiment 1). Further we investigated whether bihemispheric sensorimotor beta tACS influences bimanual motor learning (experiment 2). Experiment 1 revealed that the novel task induced learning-related improvements in performance in the absence of a speed-accuracy trade-off, with distinct learning patterns for the different types of bimanual interaction. In Experiment 2, we replicated these findings. However, against our expectations, neither in-phase nor out-of-phase beta tACS modulated learning or condition related effects.

### Novel Bimanual Motor Learning Task

We designed the novel motor task presented here to study bimanual motor learning and to characterise different levels of bimanual interaction (*Uni, Bi_*eq*_, Bi_*uneq*_*). Our results demonstrate that participants improved in both movement time and error, i.e., they learned the novel task, in the absence of a speed-accuracy trade-off. Learning-induced improvements in performance have been found previously in bimanual finger tapping (Shammi et al., 1998; Bangert et al., 2010; Takeuchi et al., 2012; Sallard et al., 2014; Koppelmans et al., 2015; Loehrer et al., 2016; Kajal et al., 2017) and more complex tasks (Preilowski, 1972; Fagard et al., 1985; Mueller et al., 2009; Sisti et al., 2011; Doost et al., 2017). Both estimates, i.e., movement time and error, can therefore be used independently to quantify bimanual motor learning in the novel task.

We explored whether prior experience with bimanual activities or achievement motivation assessed with the AMI had any influence on the learning ability for the task. However, bimanual activities and achievement motivation did not interact with learning the task in experiment 1 or 2. Whether prior experience with bimanual activities or the AMI had any influence on beta tACS cannot be answered with the current study due to the nature of the between-subject design. A within-subject design or a between-subject design with a considerable larger sample size (allowing for subgroups within stimulation groups) would be necessary to disentangle whether bimanual competence or AMI could influence the response to beta tACS.

We demonstrated a significant difference between the three conditions (unimanual, bimanual equal, and bimanual unequal). This is in line with previous literature. For instance, it has been shown that unimanual movements are easier than bimanual movements (e.g., Mueller et al., 2009). Similarly, our finding that *Bi*_*uneq*_ was more difficult than *Bi*_*eq*_ is consistent with studies investigating finger tapping or rotation tasks reporting performance in conditions that require unequal contributions between hands to be worse compared to conditions requiring equal contributions between hands, i.e., alternating tapping in different rates compared to simultaneous tapping or 3:1 rotations compared to 1:1 rotations (Fagard et al., 1985; Shammi et al., 1998; Mueller et al., 2009; Bangert et al., 2010; Sisti et al., 2011). Sisti et al. (2011) suggests that this is because the movement of the one hand needs to be “decoupled” from the other hand, which takes more effort. This is in line with the theory that movements with equal contributions of homologous body parts are generally preferred, more stable and accurate than other bimanual movements, e.g., with unequal contributions (see for review Swinnen, 2002; Swinnen and Gooijers, 2015).

Further, our results yielded distinct learning-patterns for the different levels of bimanual interaction. Specifically, for movement time learning-related changes were most pronounced for *Bi*_*uneq*_ followed by *Bi*_*eq*_ and smallest for *Uni*, whereas for error learning-related changes were most pronounced for *Bi*_*eq*,_ followed equally by *Uni* and *Bi*_*uneq*_. That the two bimanual conditions yield stronger learning-related changes than the unimanual condition might be simply because there is more room for improvement in the bimanual conditions (i.e., unimanual shows floor effect faster). One might suggest that the learning of the unimanual condition is equivalent to the learning of using the force gripper, whereby the learning of the bimanual condition comprises the additional component of bimanual interaction. Among the bimanual conditions, our results suggest that for movement time learning-related change was larger for *Bi*_*uneq*_ than for *Bi*_*eq*,_ whereby the opposite pattern was observed for error, suggesting a complex relationship.

Taken together, our novel bimanual motor learning task is suitable to study bimanual motor learning and to quantify different levels of bimanual interaction. Based on this, future electrophysiology studies can characterise intra- and interhemispheric contributions to motor networks associated with bimanual motor learning.

### Learning-Related Changes Do Not Transfer When the Hands-Axis Configuration Is Reversed

A key question for any learning study is whether the skill acquired is specific to the task trained or whether it transfers to other, similar tasks. Here, transfer of learning-related effects was explored by swapping the hand-axis configuration (i.e., transposition). Our results suggest that learning-related changes did not transfer to transposition. This might be attributed to specific aspects of both the training and the transfer tasks.

Like many motor learning studies (Preilowski, 1972; Mueller et al., 2009; Breslin et al., 2012; Doost et al., 2017), we used a small training set, i.e., small number of streets (*N* = 5; 0°, 90°, 22°, 67°, 45°), that were repeated many times (range 75–176 trials) in our training task. However, it is likely that many repetitions of a small training set reinforced the learnt association between the street angles and the required hand ratios, i.e., forming specific motor memories, rather than learning generic bimanual motor coordination. Forming motor memories instead of learning a generic motor skill has been described previously by Doost et al. (2017) and is in line with the Schema theory (Schmidt, 1975, 2003). The Schema theory defines a specific skill as an ability unique to a particular stimulus and is a result of many repetitions (Schmidt, 1975; Schmidt and Young, 1987; Wulf and Schmidt, 1997; Keetch et al., 2005; Breslin et al., 2012), whereas a generic motor skill is acquired when a given movement is abstracted as a movement representation on a higher level, e.g., sequence of individual parts of a movement, relative timing and relative force (Schmidt, 1975, 2003). In other words, in specific skills the motor memory component is relatively large and in generic motor skills the motor memory component is relatively small. We therefore hypothesise that our training task reinforced a specific skill rather than a generic skill, and that for this training task, the transfer task (transposition) was too different from the training task. In this vein, training tasks with more variability (e.g., larger number of streets) might reinforce a more general skill and thus transfer to transposition could be possible, as reported before (Shea and Kohl, 1990; Wulf and Schmidt, 1997; Breslin et al., 2012; Czyż and Moss, 2016).

Conversely, it might also be the case that our transfer task was too different from the training task for transfer to occur (Henry and Rogers, 1960; Keele, 1968; Simons et al., 2009). Studies using transfer tasks that are closely related to the training tasks, e.g., increased speed, increased distance to a target, different muscles involved, i.e., from lower limb to hand (Shea and Kohl, 1991; Keetch et al., 2005; Simons et al., 2009; Aune et al., 2017), reported transfer effects. However, transfer has not been observed when training tasks and transfer tasks are rather different, e.g., transfer from tapping to drawing, mismatch between sequences of the movement, reversal of control-display relationship, i.e., transposition (Lewis et al., 1951; Robertson et al., 1999; Grafton et al., 2002). Therefore, amending our transfer task to be closer the training task (e.g., non-trained angles, streets with corners or curves, movement direction of the cursor) might be more likely to yield transfer.

### Beta Dynamics in Bimanual Motor Learning

Experiment 2 aimed at modulating bimanual motor learning with bihemispheric in-phase and out-of-phase sensorimotor beta tACS. Specifically, we targeted both M1 to be stimulated in-phase (synchrony between both hemispheres) and out-of-phase (desynchrony between both hemispheres) and tested the effects of beta tACS on learning different levels of bimanual interactions. However, against our expectations, beta tACS did not modulate bimanual motor learning. In the following section, we will discuss several potential explanations for the absence of the hypothesised effect.

One could argue that beta was not the optimal stimulation frequency, as stimulation at 10 Hz benefited task switching in a bimanual tapping task (Heise et al., 2019) and altered motor sequence learning (Schubert et al., 2020). However, sensorimotor beta activity is relevant for bimanual motor control and can give insight into the complex intra- and interhemispheric coherence during bimanual motor learning (see for review Hummel and Gerloff, 2006; Rueda-Delgado et al., 2014). Although the role of beta activity in complex bimanual motor learning is not fully understood, interhemispheric coherence between both M1 in the beta band is present during bimanual motor learning (Serrien and Brown, 2002; Loehrer et al., 2016; Kajal et al., 2017). In addition, beta activity is different between equal and unequal contribution between hands as beta coherence shows lower interhemispheric connectivity in movements with equal contributions compared to unequal contributions (Serrien, 2008). When applying tACS with concurrent functional Magnetic Resonance Imaging, it has been shown that functional connectivity can be modulated to be in synchrony between hemispheres (Bächinger et al., 2017) and that beta activity can be changed in the targeted area (Weinrich et al., 2017). Together, it is reasonable to expect M1-M1 beta tACS to affect sensorimotor networks, communication and ultimately bimanual motor learning. As this is initial work, we cannot rule out the importance of other frequencies. Future studies investigating hemispheric synchrony during bimanual motor learning should explore the importance of frequency specificity.

Traditionally, tACS applies continuous oscillatory stimulation. However, sensorimotor beta activity is increasingly interpreted as transient high-power burst events (Jones, 2016; Shin et al., 2017; van Ede et al., 2018). The question therefore arises as to whether continuous oscillatory tACS can influence transient beta burst activity. It is likely that the effect of tACS on burst events is similar to the effect of tACS on oscillatory activity, if the burst events have an underlying rhythmic generator. Despite initial attempts to investigate whether bursts have an underlying rhythmic generator or not (Shin et al., 2017; van Ede et al., 2018; Seedat et al., 2020), this question is not finally answered. For bihemispheric tACS the case is even more complex as it is conceivable that bursts need to be coupled across hemispheres, also described as burst coincidence (Tinkhauser et al., 2020), for bihemispheric tACS to be maximally effective. However, research investigating the spatial spread of burst (Little et al., 2019; Zich et al., 2020) and burst coupling across regions (Seedat et al., 2020; Tinkhauser et al., 2020) has just begun. Together, it is not yet clear what role bursts play in the application of tACS.

A further aspect that needs to be considered is the effect of inter-regional delay. Brain hemispheres communicate via the corpus callosum to exchange cognitive and sensory information (Sperry, 1974; Ferbert et al., 1992). This interhemispheric communication has a conduction delay of 27.5–37.5 ms depending on brain mass, axon size, myelination and other inter-individual differences, i.e., age, according to *in vitro* measures (Phillips et al., 2015). Non-invasively, using transcranial magnetic stimulation (TMS), it has been suggested that bimanual contractions result in inhibition between hemispheres with an interhemispheric delay of inhibition of 6–30 ms (Ferbert et al., 1992; Fling and Seidler, 2012). During bimanual movements and bimanual motor learning, one would expect the following: Both hemispheres interact most effectively in synchrony, as underlined by the hypothesis for effective interhemispheric transfer through neuronal synchrony (Fries, 2005, 2015). Further, both hemispheres exchange information continuously and rapidly. However, the nature of our bimanual task is relatively dynamic (i.e., the contribution of both hands might vary to move the cursor as closely as possible along the ideal line). It is reasonable to assume that the interhemispheric communication is similarly dynamic. Together, this has two implications when applying M1-M1 tACS. First, the tACS effect might depend on the conduction delay. Specifically, if one assumes that the hemispheres interact most effectively when in synchrony, the conduction delay hinders an instant arrival of signals. Secondly, the continuous and rapid exchange of information between hemispheres might hinder tACS from effectively entraining the connectivity because the exchange is not entirely in-phase or out-of-phase but rapidly changing depending on the strategy of the subject. Therefore, beta tACS might not be sensitive enough for the dynamics of the task, as previously suggested in a study investigating task switching during bihemispheric in-phase tACS by Heise et al. (2019).

Finally, to fully evaluate the potential of stimulation, stimulation parameters can be optimised, e.g., by taking the inter-individual differences across participants into account. Here, we individualised the stimulation frequency. Individual beta peak frequency has been shown to be behaviourally meaningful (Kilavik et al., 2013) and stable between sessions (Espenhahn et al., 2017), but highly variable across participants, which emphasises the need to individualise stimulation frequency. This is in line with the findings that the effects of tACS are more pronounced when stimulation frequency matches the natural, individual frequency (Zaehle et al., 2010; Thut et al., 2011; Ali et al., 2013; Reato et al., 2013; Schilberg et al., 2018). In addition to the tACS frequency, phase has been reported to influence motor performance in unilateral tACS (Polanıa et al., 2012; Violante et al., 2017), in-phase bihemispheric tACS (Heise et al., 2019) and functional connectivity in a modelling study (Schwab et al., 2019). However, to stimulate tACS in-phase, electrophysiological data are preferably recorded simultaneously (Helfrich et al., 2014b; Neuling et al., 2017). While this approach is technically very demanding, it uniquely enables the measurement of tACS-related neural changes during and after stimulation.

Conventionally, non-invasive brain stimulation is applied at a fixed amplitude (e.g., 2 mA). However, a fixed-amplitude stimulation does not account for inter-individual differences in neuroanatomy, such as skull thickness, which are likely to lead to varying amplitude entering the brain across individuals. To achieve a comparable amplitude at brain level across individuals, stimulation amplitude could be individualised. Indeed, amplitude-controlled stimulation has been described to reduce the variability of current intensities, as well as the distribution of the electric field across individuals (Evans et al., 2020). Another way to account for inter-individual differences in tACS is to optimise the electrode montage. Individualising the electrode montage guided by structural Magnetic Resonance Imaging allows to achieve optimised current flow and direction while maintaining a high electrical field intensity above the target area (Lee et al., 2020). This can be further optimised in combination with differently sized electrodes and high-definition 4-1 montages (Helfrich et al., 2014a) as well as ring electrodes (Heise et al., 2016) as this stimulation is more focal. However, those montages come with their own disadvantages, e.g., amongst others, a significantly weaker electric field over the stimulation target (see for a review Riddle and Frohlich, 2021).

Optimising the stimulation parameters as discussed above could help to fully evaluate the potential of bihemispheric stimulation in bimanual motor learning although their advantages should be balanced against their disadvantages and technical challenges.

### Limitations

In this study, most participants reported seeing phosphenes during active tACS. We warned participants that they might see phosphenes depending on their individual reaction to the stimulation, and although sensory side effects like phosphenes are unlikely to hamper the physiological effect of tACS (Schwab et al., 2019), participant’s performance might have been influenced indirectly through attentional bias or discomfort.

### Conclusion

The novel bimanual motor task allowed to characterise bimanual motor learning with different levels of bimanual interaction. This was replicated in an independent sample. Here, neither in-phase nor out-of-phase beta tACS modulated the learning-related changes. Investigating the neural mechanisms underlying bimanual motor learning will provide new insights and enable more targeted modulation of brain activity to improve complex bimanual motor learning.

## Data Availability Statement

Data, the code for the task, and the code for data analysis might be shared upon request to the corresponding author.

## Ethics Statement

The studies involving human participants were reviewed and approved by the University of Oxford; MSD-IDREC-R61309/RE001 and University of Oxford; MSD-IDREC-R65118/RE001. The patients/participants provided their written informed consent to participate in this study.

## Author Contributions

CZ: conceptualisation, methodology, supervision, and writing – review and editing. MS: conceptualisation, methodology, investigation, formal analysis, and writing – original draft. CS: conceptualisation, methodology, resources, funding acquisition, supervision, and writing – review and editing. I-FG: investigation, managing double-blinding procedure, and writing – review and editing. All authors contributed to the article and approved the submitted version.

## Conflict of Interest

The authors declare that the research was conducted in the absence of any commercial or financial relationships that could be construed as a potential conflict of interest.

## Publisher’s Note

All claims expressed in this article are solely those of the authors and do not necessarily represent those of their affiliated organizations, or those of the publisher, the editors and the reviewers. Any product that may be evaluated in this article, or claim that may be made by its manufacturer, is not guaranteed or endorsed by the publisher.

## Footnotes

1
www.emotive.com

2
www.easycap.de
