# Supplementary material for: Investigating Different Levels of Bimanual Interaction With a Novel Motor Learning Task: A Behavioural and Transcranial Alternating Current Stimulation Study
Source: Front Hum Neurosci. 2021 Nov 16;15:755748. doi: 10.3389/fnhum.2021.755748 (PMC8635148; doi:10.3389/fnhum.2021.755748)
Supplement: Supplementary file 1 [file Table_1.DOCX]

Supplementary Material

Supplementary Table 1 – Results of the 2 x 2 ANOVA with a within-subject factor of task (training and transposition) and timepoint (before and after learning the training task) with movement time and error as dependent variables.

|  | df | Movement time | | | Error | | |
| --- | --- | --- | --- | --- | --- | --- | --- |
|  |  | F | p | n^2^_p_ | F | p | n^2^_p_ |
| Task | 1,39 | 17.71 | <0.001 | 0.312 | 49.681 | <0.001 | 0.560 |
| Timepoint | 1,39 | 36.465 | <0.001 | 0.483 | 0.972 | 0.33 | 0.024 |
| Task x Timepoint | 1,39 | 26.003 | <0.001 | 0.400 | 107.683 | <0.001 | 0.734 |
